# Native mass spectrometry interrogation of complexes formed during targeted protein degradation

**DOI:** 10.1002/rcm.9604

**Published:** 2023-09-18

**Authors:** Ikhlas M. M. Ahmed, Rebecca Beveridge

**Affiliations:** ^1^ Department of Pure and Applied Chemistry University of Strathclyde Glasgow UK

## Abstract

**Rationale:**

Protein degraders are small molecules that promote cellular degradation of a target protein. Degraders simultaneously bind to their target and an E3 ligase, bringing them into close spatial proximity, but the formation of this ternary complex is difficult to measure with many biophysical techniques.

**Methods:**

Native mass spectrometry (nMS) is an effective label‐free technique to identify the complexes formed by proteolysis‐targeting chimeras (PROTACs). It can monitor the formation of ternary E3–PROTAC–target complexes and detect intermediate binary species. Experiments are described using a Synapt G2Si (Waters) equipped with a nano‐electrospray ionisation source.

**Results:**

The protocol describes nMS experiments for measuring the complexes formed by PROTAC molecules. It also describes how to investigate differences in the affinity of PROTAC complexes, whether a PROTAC shows specificity for a given target and whether a PROTAC shows cooperative behaviour.

**Conclusions:**

Here, we provide step‐by‐step instructions for the sample preparation of PROTAC complexes and their nMS interrogation to obtain optimal information on their binding modes.

## 1 INTRODUCTION

Proteolysis‐targeting chimeras (PROTACs) are small‐molecule degraders that eliminate target proteins by programming them for degradation by the cell.^1^, ^2^ PROTACs are bifunctional molecules that consist of two ligands joined by a linker region.^3^ The ligands are specific for a target protein and an E3 ligase respectively.^4^ The PROTAC therefore brings the two proteins into close spatial proximity, causing the E3 ligase to catalyse ubiquitination of the target protein, thereby labelling it for proteasomal degradation by the cell.^5^ The term ‘target protein’, which is also referred to as the ‘substrate’ or ‘protein of interest’, will be used throughout the text.

A main challenge in studying PROTACs is the ternary binding system that involves the E3 ligase, the target protein and the PROTAC. Many biochemical methods have been developed to measure binary systems, often requiring adaptations and approximations to study ternary complexes. Such methods include surface plasmon resonance, isothermal titration calorimetry and size exclusion chromatography.^6^, ^7^, ^8^ Native mass spectrometry (nMS) is a highly applicable method to study protein complexes formed by PROTACs as it reports on multiple binding stoichiometries present in dynamic protein mixtures, including species populated to a low extent. This sensitive, label‐free method can be applied to proteins of varying mass and polydispersity, i.e. different shapes and sizes. Protocols detailing nMS experiments are available that describe analysis of large protein complexes,^9^ analysis of monoclonal antibodies^10^ and direct characterisation of overproduced proteins,^11^ among others. We have recently demonstrated that nMS can report on the formation of ternary complexes, determine the binding specificity of a PROTAC and compare complex formation with multiple target proteins in a single measurement.^12^

Whilst this protocol was developed for PROTACs, it is suitable for the analysis of other protein degraders such as molecular glues^13^, ^14^ (as demonstrated by Bellamy‐Carter et al^15^), SNIPERS^16^ and other bifunctional molecules.^17^

### 1.1 Materials

1. Analytical balance (Fisher Scientific catalogue no. 15907500)
2. Fisherbrand™ accuSpin™ Micro 17 microcentrifuge (Fisher Scientific catalogue no. 13‐100‐675)
3. Graduated cylinders, 10–500 mL
4. Microcentrifuge tubes 1.5 mL (Fisher Scientific catalogue no. 05‐408‐129)
5. Desalting columns, Bio‐Spin® P‐6 gel columns, Tris buffer (BIO‐RAD catalogue no.7326227)
6. 96‐well microdialysis plate, 3.5 MWCO (Thermo Scientific catalogue no. 88262)
7. Metal spatula
8. Deionised water (Fisher Scientific catalogue no. 23‐751610)
9. Pipettes (Fisher Scientific catalogue no. 14‐388‐100)
10. Pipette tips, variable from 0.1 to 1000 μL
11. Gel loader tip (Eppendorf catalogue no. 0030001222)
12. Mass spectrometer for native protein studies (examples of commercially available instruments and their corresponding recommended settings are described in Section 2.7)
13. Nano‐electrospray emitters. We recommend using metal‐coated borosilicate capillaries (Thermo Scientific part number ES380).
14. UV–visible spectrophotometer (optional; see Section 2.1.3) (Varian Cary 50 UV–Vis spectrophotometer)
15. UV–visible cuvettes (optional; see Section 2.1.3) (Fisher Scientific catalogue no. 14‐955‐127)
16. Nanodrop (optional; see Section 2.1.3) (Thermo Scientific NanoDrop 1000)
17. Dry wipes (Fisher Scientific catalogue no. 06‐666A)

### 1.2 Chemicals

1. Ammonium acetate (Thermo Scientific catalogue no. A16343.30)
2. Dimethylsulfoxide (DMSO) LC/MS grade (Thermo Scientific catalogue no. 85190)
3. PROTACs
4. Purified E3 ligase
5. Purified target proteins
6. Bovine serum albumin (BSA) (optional; see Section 2.1.3) (Sigma‐Aldrich catalogue no. A2153)
7. Myoglobin (Sigma‐Aldrich catalogue no. M0630)
8. Leu‐enkephalin (Leu‐Enk) acetate salt from Bachem (Fischer Scientific catalogue no. 50‐259‐648)
9. Bradford reagent (optional; see Section 2.1.3) (Thermo Scientific catalogue no. 23238)

## 2 METHOD

### 2.1 Buffer exchange proteins into ammonium acetate/dissolve PROTAC in DMSO

Ammonium acetate is normally the solution of choice for nMS experiments, as it is highly volatile and hence evaporates from the proteins readily during desolvation. As the PROTAC will likely be dissolved in DMSO or another organic solvent, ensure that the proteins can be analysed in the presence of this and be aware of any alterations that it confers to the protein signal (i.e. charge reduction in the presence of DMSO^18^, ^19^). Moreover, keep the final DMSO/organic solvent concentration as low as possible; in this protocol the DMSO concentration is kept at 1% and it is not recommended to exceed 5%.

#### 2.1.1 Prepare ammonium acetate solution (200 mM, 50 mL); 15–30 min

**Table:** 

Time	Step	Comments/tips
15–30 min	Measure out 770 mg in a 50 mL conical centrifuge tube. Add 50 mL of deionised water to conical centrifuge tube. Shake until ammonium acetate is fully dissolved.	If MS signal is low for the proteins, the concentration of ammonium acetate could be altered to achieve optimal solution conditions. Ammonium bicarbonate can also be used as an alternative solution.

#### 2.1.2 Buffer exchange proteins into ammonium acetate

**Table:** 

Time	Step	Comments/tips
Micro bio spin chromatography columns		
15–30 min	Invert the column sharply several times to resuspend the settled gel and remove any bubbles. Snap off the tip and place the column in a 2.0 mL microcentrifuge tube (included). Now remove the top cap. If the column does not begin to flow, push the cap back on the column and then remove it again to start the flow. Allow the excess packing buffer to drain by gravity to the top of the gel bed (about 2 min). Discard the drained buffer then place the column back into the 2.0 mL tube. Centrifuge for 2 min in a microcentrifuge at 1000 × *g* (see Centrifugation Notes section) to remove the remaining packing buffer. Discard the buffer. Apply the new buffer in 500 μL aliquots. After each application of new buffer, let the buffer drain out by gravity, or centrifuge the column for 1–2 min to remove the buffer. Discard buffer from collection tube. Repeat as required. Three washes result in >99% of the buffer exchanged. Four washes result in >99.9% of buffer exchanged. Place the column in a clean 1.5 or 2.0 mL microcentrifuge tube. Carefully apply the sample (20–75 μL) directly to the centre of the column. Application of more or less than the recommended sample volume may decrease column performance. After loading sample, centrifuge the column for 4 min at 1000 × *g*. Following centrifugation, the purified sample is now in ammonium acetate solution. Molecules smaller than the column's exclusion limit will be retained by the column (see specifications of column manufacturer).	These instructions are adapted from the instruction manual for micro Bio‐Spin Chromatography columns, which can be found on bio‐rad.com .

If there is a severe loss of protein concentration when using the spin columns, or if the protein target is intrinsically disordered and/or prone to aggregation, then microdialysis devices can be used as an alternative.

**Table:** 

Time	Step	Comments/tips
Microdialysis		
2 h	Remove one or more microdialysis devices; the round opening is used to introduce and remove buffer/sample (handle device with gloves). Add 100 μL dialysis buffer, then remove it (do not let membrane go dry). Load sample (10–100 μL) making sure the sample is at the bottom of the device. Place device into a 2 mL microcentrifuge tube containing 1400 μL of dialysis buffer (ammonium acetate) on ice. Dialyse for 2 h by moving the device into fresh ammonium acetate every 20 min for the first hour, then every 30 min for the second hour. Recover the sample (now in desired buffer) from the round opening. (Optional) Centrifuge for 5 min at 10 000 × *g* to remove any aggregates. Following dialysis, the purified sample is now in ammonium acetate solution. Molecules smaller than the device's exclusion limit will be in the dialysis buffer (see specifications).	These instructions are adapted from the instruction manual for Pierce 96‐well microdialysis plate, which can be found on thermofisher.com.

#### 2.1.3 Measuring protein concentration with Bradford assay or nanodrop

##### Bradford assay

**Table:** 

Time	Step	Comments/tips
45–60 min	Dissolve 0.5 mg of BSA in 0.5 mL of water to prepare 1 μg/μL BSA. Prepare standards in microcentrifuge tube (1.5 mL) as follows: **Table:** Water (mL) BSA, 1 μg/μL (μL) Bradford reagent (mL) Blank 1 — — BSA 1 μg/mL 0.5 1 0.5 BSA 2 μg/mL 0.5 2 0.5 BSA 4 μg/mL 0.5 4 0.5 BSA 6 μg/mL 0.5 6 0.5 BSA 8 μg/mL 0.5 8 0.5 BSA 10 μg/mL 0.5 10 0.5 Estimate sample protein volume for 1, 5 and 10 μg. Protein dilution might be required. Add the appropriate volumes of sample protein to 0.5 mL water/0.5 mL Bradford reagent. Mix by inverting the microcentrifuge tube, and transfer samples to disposable UV–visible cuvettes. Measure absorbance at 595 nm using blank for zeroing the reading. Prepare a scatter graph for BSA standards: *A*_595nm_ (*Y*‐axis) versus BSA mass/μg (*X*‐axis). The *R* ^2^ value should be above 0.9; record the *m* and *c* values from the graph (*y* = *mx* + *c*). Concentration of sample (mg/mL) = [(sample absorbance + (−*c*)/*m*)/(μL of added protein sample)]. Take average of all three readings for final concentrations.	Ensure the samples are measured quickly (within 30 min of the addition of the reagent) as the method is time‐sensitive. The absorbance readout for the samples should be within the calibration range (i.e. 1–10 μg). If not, the concentration assay will need to be repeated with diluted samples.

##### Nanodrop

**Table:** 

Time	Step	Comments/tips
15–30 min	Using the protein A280 option, initialise the nanodrop by adding 1–2 μL water. Blank with 1–2 μL solution (ammonium acetate). Apply 1–2 μL of sample to obtain absorbance at 280 nm. Use the molar extinction coefficient of the protein to estimate the concentration.	Avoid air bubbles as they affect the measurement. Wipe the sample surface and the cover after each step with delicate wipes. Always start and end with a water measurement. Calculate the molar extinction coefficient from the primary sequence (manually) or by using the online ProtParam tool from Expasy (https://web.expasy.org/protparam/). In Beveridge et al,^12^ sequences for ternary complex components are provided.

#### 2.1.4 Prepare PROTAC solution

**Table:** 

Time	Step	Comments/tips
15–30 min	Add DMSO to PROTAC to make a concentration of 10 mM. This will be the stock solution. Add 1 μL of stock solution to 99 μL of deionised water (100 μM PROTAC in 1% DMSO). Dilute to 2× working concentration with 1% DMSO to ensure constant DMSO concentration across all experiments.	If the PROTAC is soluble in a solvent other than DMSO, this can be used instead. At each step, check that the PROTAC is dissolved by looking at it under a light microscope. If solubility problems occur, an assay can be set up to determine the best solvent to use. The concentration of organic solvent should be kept constant across all experiments.

### 2.2 Protein quality control and determination of optimum protein concentrations

This step is to measure the mass of the individual proteins and assess their purity.

#### 2.2.1 Analyse the proteins individually for quality control, to optimise concentrations and to measure their mass

**Table:** 

Time	Step	Comments/tips
45–60 min per sample	Dilute the protein to 10 μM with 200 mM ammonium acetate, then dilute 2× with deionised water to give a protein concentration of 5 μM and ammonium acetate concentration of 100 mM. Analyse the samples using nano‐electrospray (nESI) MS which requires low sample volumes, low concentrations and is a soft ionisation method. We recommend using metal‐coated borosilicate capillaries (Thermo Scientific). Example settings for the mass spectrometer are provided in Section 2.7. Determine the isotope‐averaged molecular mass of the proteins, using a deconvolution software such as Origami^20^ or Unidec.^21^	For nESI‐MS, 10 μL per sample is required. If signal intensity is low, try increasing or decreasing the protein concentration. Decreasing the protein concentration can have a positive effect on the signal intensity, as competitive ionisation can occur at higher concentrations.^22^

### 2.3 Test for ternary and binary complex formation

**FIGURE 1:**
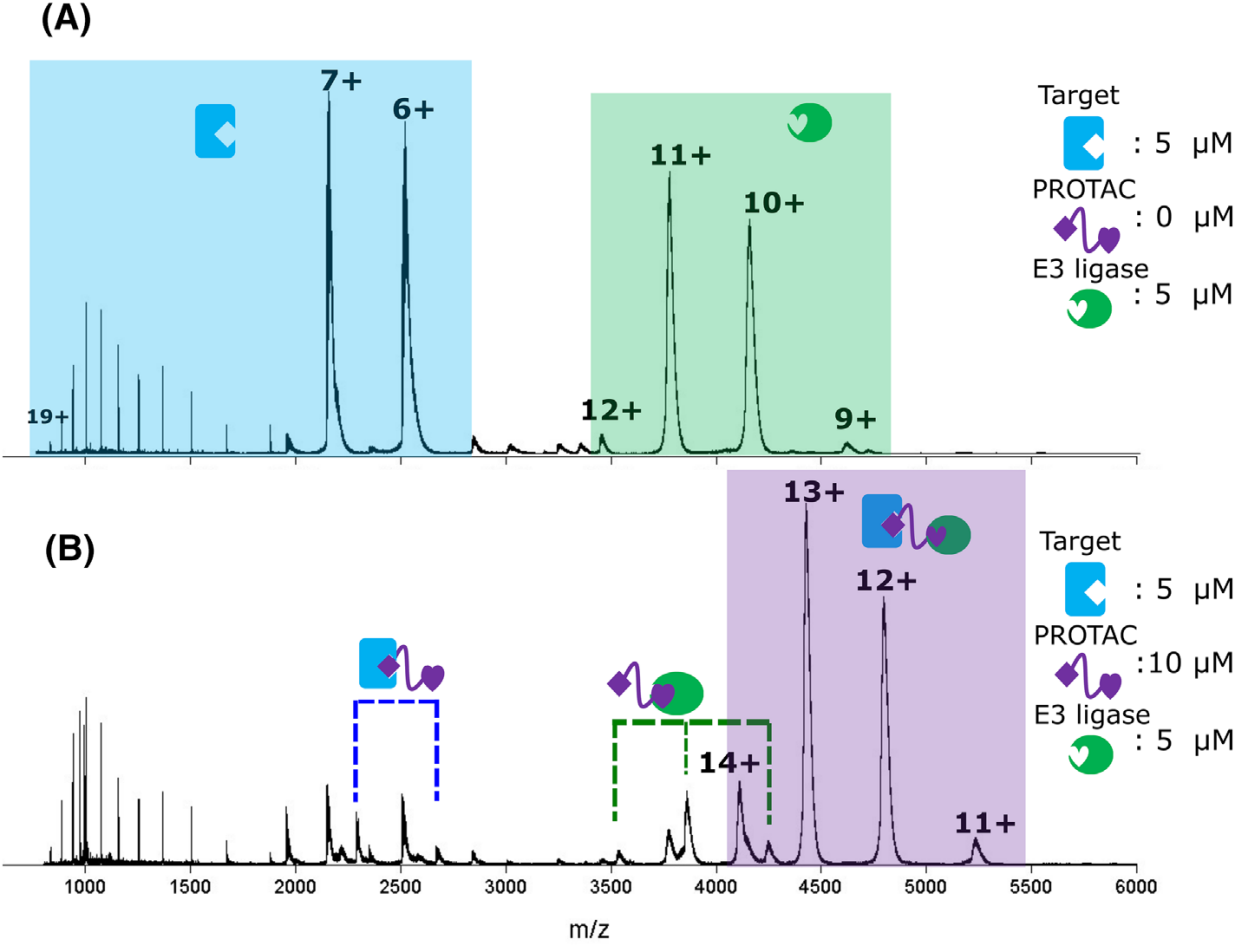
Characterising the binding equilibrium between the E3 ligase (5 μM), the proteolysis‐targeting chimera (PROTAC) and the target (5 μM) by nMS in the absence of PROTAC (A) and in the presence of PROTAC (10 μM) (B). Figure adapted from Beveridge et al.^12^ [Color figure can be viewed at wileyonlinelibrary.com]

This step is to identify whether ternary complexes are formed with a given E3–PROTAC–target combination. Identification of which charge states the complex is present in (see Data S1) will also help with data analysis at further points in the protocol (e.g. Section 2.6). In these spectra, the presence of binary interactions (PROTAC–E3 and PROTAC–target) in the reaction mixture can also be identified. Figure 1A shows an example mass spectrum of a target and E3 in the absence of PROTAC, where no interaction occurs. Here, the target protein, highlighted in blue, is present in charge states 6+ to 19+, with most of the intensity in the 6+ and 7+ charge states. The E3 ligase, highlighted in green, is present in charge states 9+ to 12+. Figure 1B shows a spectrum of the same target and E3 as in Figure 1A, with the addition of 10 μM PROTAC. In this case, the ternary complex (highlighted in pink) and target–PROTAC and E3–PROTAC binary complexes are observed.

**Table:** 

Time	Step	Comments/tips
15–30 min	From measuring the average isotopic mass of each protein, calculate the predicted mass and *m*/*z* ratios of binary interactions between each protein and the PROTAC (E3–PROTAC complex and PROTAC–target complex), between both proteins without the PROTAC (E3–target) and of the ternary complex (E3–PROTAC–target) (see supporting information). Analyse both proteins in a mixture at the determined optimum concentration and determine whether the proteins interact in the absence of PROTAC. Add PROTAC at varying concentrations (0.25×, 0.5×, 1×, 2×, 5× protein concentration), and annotate peaks identified in the spectra. Optimise experimental conditions including best protein and PROTAC concentrations for ternary complex formation.	In previous work,^12^ a 5:10:5 μM concentration of E3:PROTAC:target was found to be optimal for ternary complex formation. Look out for hook effect at high PROTAC concentration. This is the preference of binary complex formation with each protein over the formation of ternary complexes. Data S1 provides calculated charge states of a single protein, binary complex and ternary complex. As an initial step in identifying individual components of the ternary complex, native mass spectra can be deconvoluted using specific software (mentioned in Section 2.2.1). Peaks representing ternary complex formation or higher‐order species may be incorrectly assigned during deconvolution due to the complexity of these data; therefore, the use of the provided spreadsheet alongside deconvolution software is strongly recommended for correct assignment of peaks.

### 2.4 Test for specificity

**FIGURE 2:**
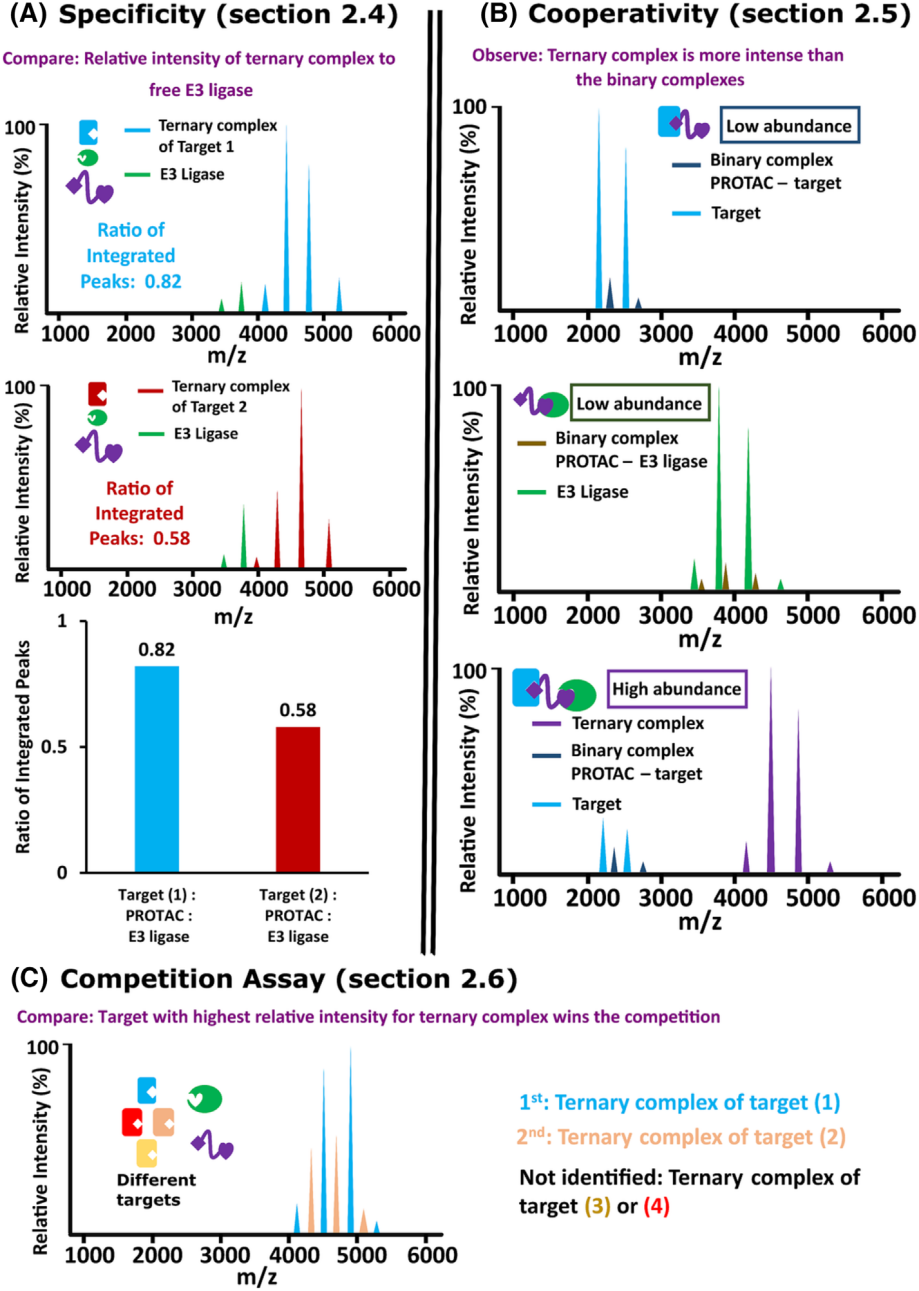
nMS for specificity (A), cooperativity (B) and competition assay studies (C). [Color figure can be viewed at wileyonlinelibrary.com]

PROTACs can recruit closely related target proteins to different extents,^7^ and the specificity of a PROTAC can be estimated by nMS by comparing the relative intensity of the ternary complex to that of the unbound E3 ligase (Figure 2A).

**Table:** 

Time	Step	Comments/tips
15–30 min	Upon determination of successful conditions to observe a ternary complex, different potential target proteins can be tested to observe which is most preferentially recruited to the E3 ligase by a particular PROTAC. Measure each target protein with the optimised E3 and PROTAC concentration and identify peaks that correspond to the binary and ternary complexes. A higher relative signal intensity of the ternary complex compared to the unbound E3 ligase indicates target preference.	Running these experiments using different PROTAC concentrations would reveal additional information regarding the selectivity towards a target; e.g. this may only be differentiated at low PROTAC concentrations.

### 2.5 Test cooperativity

In a cooperative system, the ternary complex will form more readily than either of the binary complexes, which is thought to contribute to the specificity of the PROTAC. Cooperativity can be measured by comparing binary complex formation to ternary complex formation. In a cooperative system, binary complexes between the PROTAC and either protein will be low (Figure 2B).

**Table:** 

Time	Step	Comments/tips
15–30 min	Analyse target protein–PROTAC and E3–PROTAC separately to measure binary complex formation. Compare ternary complex formation to binary complex. If the signal intensity for ternary complexes is higher than for binary complexes, the ternary complex formation is likely cooperative.	For in‐depth study, analyse binary complex (PROTAC–target or PROTAC–E3) in the presence of increasing concentrations of the third component and monitor the ternary complex formation.

### 2.6 Develop competition assays

To take full advantage of the ability of nMS to measure complex reaction mixtures, assays can be developed in which the specificity for multiple target proteins can be measured in a single experiment. Measuring the target proteins in mixtures is more time‐effective than separate measurements and has the added advantage of providing information on competition between targets forming the ternary complexes (Figure 2C).

**Table:** 

Time	Step	Comments/tips
15–30 min	Analyse a mixture of potential targets at equal concentration to the E3 ligase, with the optimal amount of PROTAC identified in Section 2.3, to identify which targets are recruited when there is no competition for binding. To measure which targets win competition for binding, increase the concentration of the targets relative to the E3 ligase. Higher relative signal intensity indicates the preferred target.	Use a selective versus unselective PROTAC to determine ternary complex formation preference under each condition and the preferred target.

### 2.7 Example instrument settings

The following tables provide published non‐default/tuneable settings for nMS studies on ternary complexes. Adjustable parameters are both sample and instrument specific which can be tuned to produce optimum results. We suggest these settings as a starting point for experiments.

#### 2.7.1 Example source parameters for Synapt G2Si (Waters), taken from Beveridge et al^12^

**Table:** 

Capillary voltage	1.1–1.3 kV
Sample cone	40–80 V
Source offset	30–100 V
Ion mobility spectrometry (IMS) bias voltage	2 V
Source temperature	40°C
Trap gas flow	2–3 mL/min

#### 2.7.2 Example instrument parameters for Q‐exactive HF (Thermo Fisher Scientific) with Triversa NanoMate (Advion) taken from Bellamy‐Carter et al^15^

**Table:** 

Ionisation voltage	1.75 kV
Gas pressure	0.3 psi
Source temperature	250°C
In‐source dissociation	Off
S‐lens radiofrequency (RF)	100
Maximum ion injection time	100 ms
Automatic gain control	1 × 10^6^
Resolution	15 000

## 3 QUALITY CONTROL

To ensure good working condition of the mass spectrometer, standards are infused and the intensity is monitored. For example, Leu‐Enk (1 ng/μL in 50:50 (v/v) acetonitrile–water + 0.1% formic acid) should give signal intensity of >1e7, myoglobin (5 μM in 100 mM ammonium acetate) should give signal intensity of >1e5 and BSA (5 μM in 100 mM ammonium acetate) should give signal intensity of >1e4 using our Waters Synapt G2Si (Figures S1–S3).

### PEER REVIEW

The peer review history for this article is available at https://www.webofscience.com/api/gateway/wos/peer-review/10.1002/rcm.9604.

## Supporting information


**Data S1.** Supporting information.

**Figure S1.** nESI‐MS of Leu‐Enk (1 ng/ul) in 50:50 acetonitrile/water + 0.1% Formic Acid.
**Figure S2.** nESI‐MS of Myoglobin (5 μM) in ammonium acetate (100 mM).
**Figure S3.** nESI‐MS of BSA (5 μM) in ammonium acetate (100 mM), charge states [M+21H]^21+^ to [M+23H]^23+^ correspond to a dimer.

## Data Availability

Data sharing not applicable ‐ no new data generated.
