# Supplementary material for: Native mass spectrometry interrogation of complexes formed during targeted protein degradation
Source: Rapid Commun Mass Spectrom. 2023 Sep 18;37(22):e9604. doi: 10.1002/rcm.9604 (PMC10909470; doi:10.1002/rcm.9604)
Supplement: Supplementary file 2 — Figure S1. nESI‐MS of Leu‐Enk (1 ng/ul) in 50:50 acetonitrile/water + 0.1% Formic Acid. Figure S2. nESI‐MS of Myoglobin (5 μM) in ammonium acetate (100 mM). Figure S3. nESI‐MS of BSA (5 μM) in ammonium acetate (100 mM), charge states [M+21H]21+ to [M+23H]23+ correspond to a dimer. [file RCM-37-e9604-s002.docx]

**Quality control:**


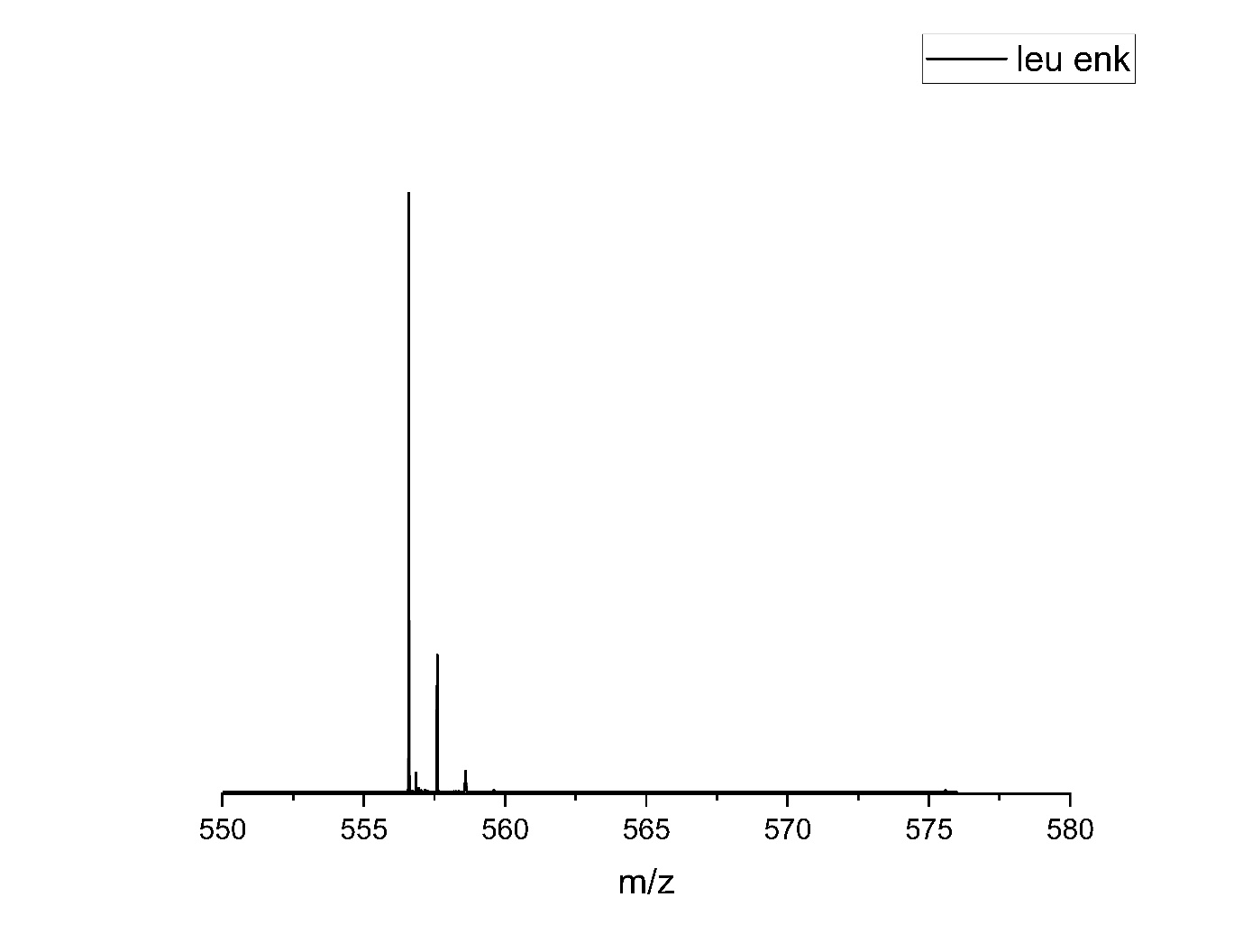


**Supplementary information Figure 1.** nESI-MS of Leu-Enk (1ng/ul ) in 50:50 acetonitrile/water + 0.1% Formic Acid.

| Sample cone | 20V |
| --- | --- |
| Source offset | 30V |
| IMS Bias voltage | 2V |
| Source temperature | 40°C |
| Trap gas flow | 2 mL/min |
| Lock spray flow control (fluidics) | |
| Capillary voltage | 2 kV |
| Flow rate | 1.8 µl/min |


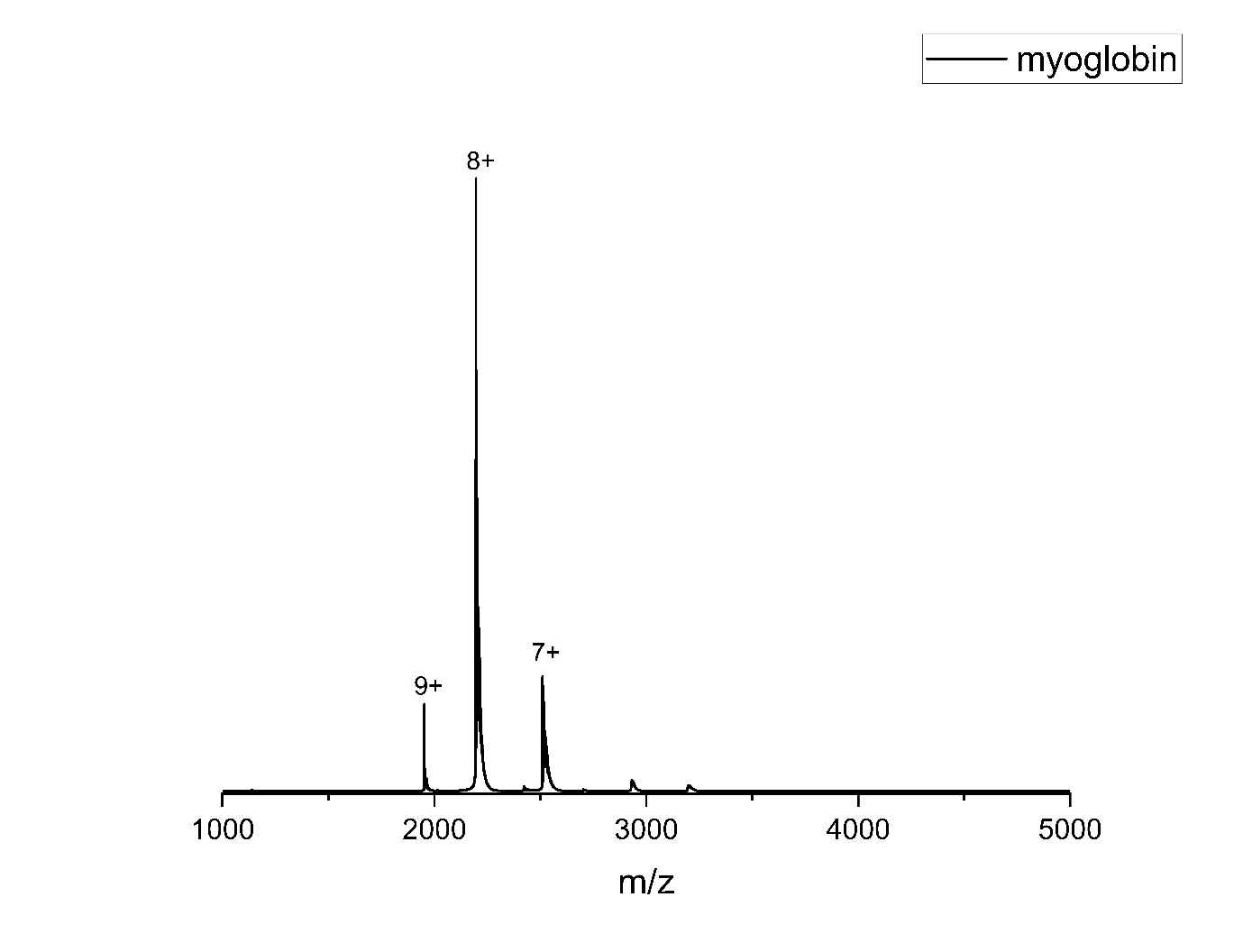


**Supplementary information Figure 2.** nESI-MS of Myoglobin (5 µM) in ammonium acetate (100 mM)

| Capillary voltage | 1.2 kV |
| --- | --- |
| Sample cone | 100V |
| Source offset | 120V |
| IMS Bias voltage | 2V |
| Source temperature | 40°C |
| Trap gas flow | 2 mL/min |


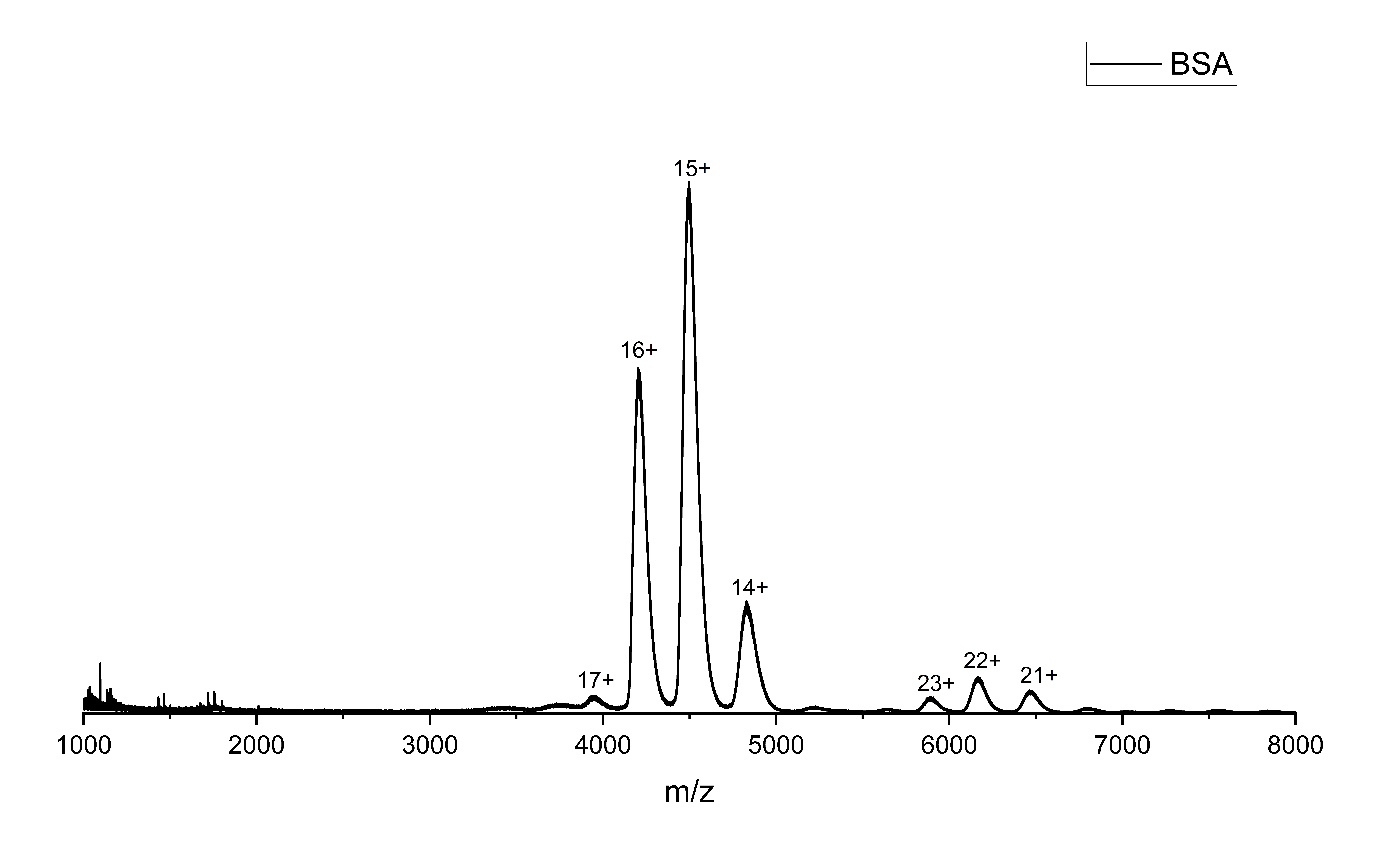


**Supplementary information Figure 3.** nESI-MS of BSA (5 µM) in ammonium acetate (100 mM), charge states [M+21H]^21+^ to [M+23H]^23+^ correspond to a dimer.

| Capillary voltage | 0.98 kV |
| --- | --- |
| Sample cone | 100V |
| Source offset | 120V |
| IMS Bias voltage | 2V |
| Source temperature | 40°C |
| Trap gas flow | 3.5 mL/min |
